# Design of micromagnetic arrays for on-chip separation of superparamagnetic bead aggregates and detection of a model protein and double-stranded DNA analytes

**DOI:** 10.1038/s41598-021-84395-3

**Published:** 2021-03-05

**Authors:** Stefano Rampini, Peng Li, Dhruv Gandhi, Marina Mutas, Ying Fen Ran, Michael Carr, Gil U. Lee

**Affiliations:** 1 School of Chemistry, University College Dublin, Belfield, Dublin, Ireland; 2 Conway Institute for Biomolecular and Biomedical Research, University College Dublin, Dublin, Ireland; 3 National Virus Reference Laboratory, University College Dublin, Belfield, Dublin, Ireland; 4 Global Institution for Collaborative Research and Education (GI-CoRE), Hokkaido University, Kita-ku, Sapporo, Japan

**Keywords:** Assay systems, Nanobiotechnology, Biotechnology, Pathogenesis, Nanoscience and technology

## Abstract

Magnetically actuated lab-on-a-chip (LOC) technologies have enabled rapid, highly efficient separation of specific biomarkers and cells from complex biological samples. Nonlinear magnetophoresis (NLM) is a technique that uses a microfabricated magnet array (MMA) and a time varying external magnetic field to precisely control the transport of superparamagnetic (SPM) beads on the surface of a chip based on their size and magnetization. We analyze the transport and separation behavior of SPM monomers and dimers on four MMA geometries, i.e., circular, triangular, square and rectangular shaped micromagnets, across a range of external magnetic field rotation frequencies. The measured critical frequency of the SPM beads on an MMA, i.e., the velocity for which the hydrodynamic drag on a bead exceeds the magnetic force, is closely related to the local magnetic flux density landscape on a micromagnet in the presence of an external magnetic field. A set of design criteria has been established for the optimization of MMAs for NLM separation, with particular focus on the shape of the micromagnets forming the array. The square MMA was used to detect a model protein biomarker and gene fragment based on a magnetic bead assembly (MBA) assay. This assay uses ligand functionalized SPM beads to capture and directly detect an analyte through the formation of SPM bead aggregates. These beads aggregates were detected through NLM separation and microscopic analysis resulting in a highly sensitive assay that did not use carrier fluid.

## Introduction

Superparamagnetic (SPM) microparticles have been used to efficiently separate nucleic acids, proteins, viruses, and cells from complex samples for lab-on-a-chip (LOC) sensing, e.g., SPM beads functionalized with antibodies can be used to isolate as few as 100 copies of a virus from a milliliter of serum sample in 2 min^1,2^. Magnetophoretic LOC research initially focused on miniaturizing high gradient magnetic field separation due to the central role SPM beads play in in vitro diagnostics^3,4^, and has rapidly expanded to include a number of functions, including simultaneous detection of multiple analytes^5–7^. Significantly, on-chip detection technologies have been developed to count SPM beads using giant magnetoresistance^8^, tunneling magnetoresistance^9^, planar Hall magnetoresistance^10^, and optical sensing^11^. The integration of high gradient separation with these sensors promises to deliver devices that are capable of rapid and sensitive point of care testing (POCT)^12–14^.

**Figure 1:**
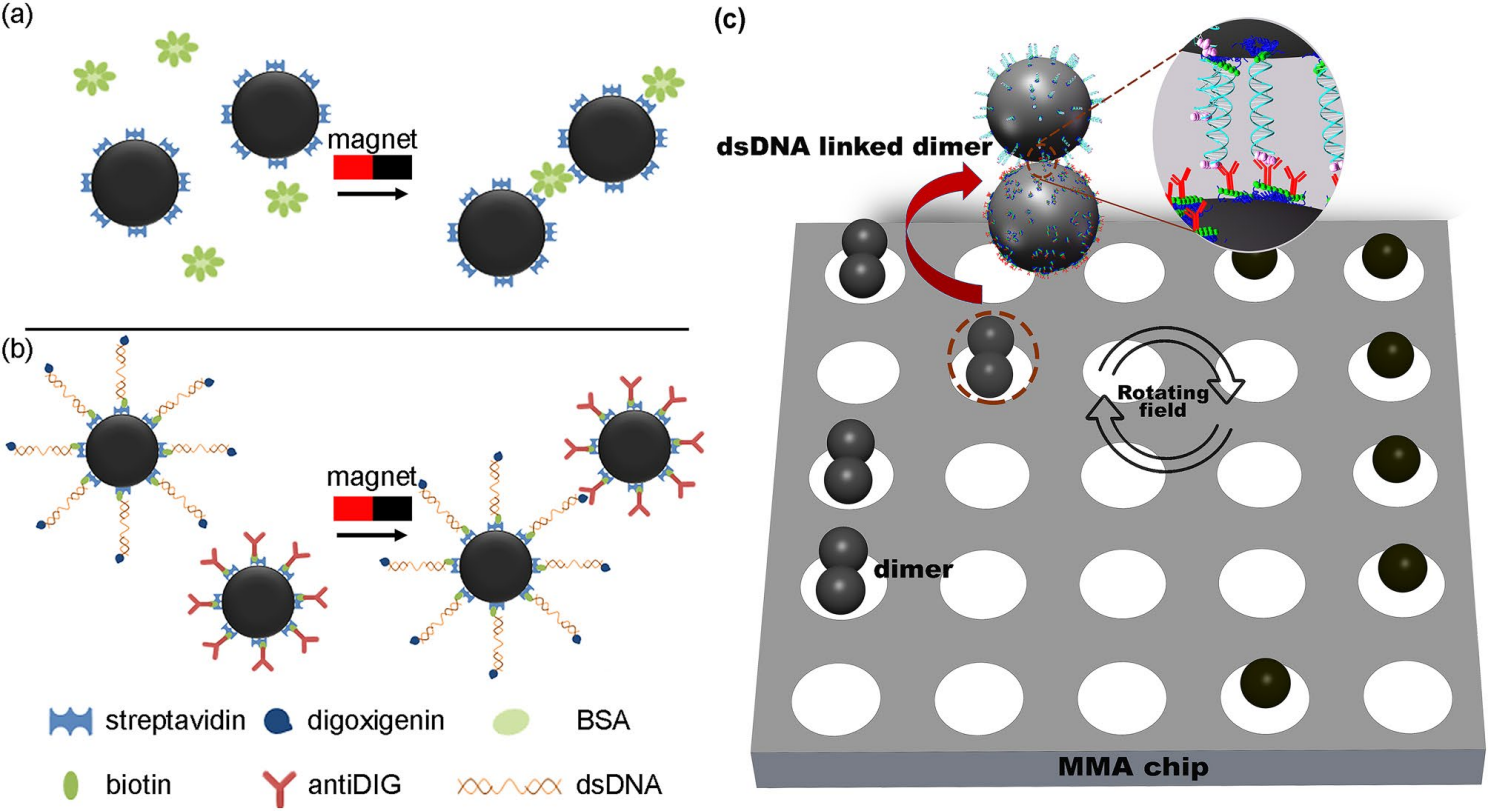
Schematics of the SPM bead chemistries used to detect biotinylated bovine serum albumin and double-stranded DNA. (**a**) Biotinylated bovine serum albumin works as a linker between streptavidin coated 2.8 μm SPM beads. A permanent magnet is used to bring the beads in close contact with each other, thus enhancing the probability of binding. (**b**) Streptavidin coated 2.8 μm SPM beads bind to double-stranded DNA (dsDNA) from HSV-1 with biotin on 3′ end. The dsDNA was also modified with digoxigenin on 5′ end to bind with 2.8 μm SPM beads coated with an antidigoxigenin antibody. (**c**) Illustration of NLM separation of dsDNA linked SPM beads aggregates from single SPM beads on a NLM chip in a rotating magnetic field.

Single-step detection of an analyte can be achieved by monitoring the assembly of SPM beads^2,11,15,16^. A key feature of SPM microparticles is that particle–particle interactions are minimal in the absence of a significant applied magnetic field and thus rapid rates of mass transfer and reaction take place in dense microparticle suspensions^17^. Nonmagnetic particle aggregation induced by cross-reaction does not take place rapidly due to the repulsive hydrodynamic force inherent in these suspensions. In the magnetic bead assembly (MBA) assay, a high gradient magnetic field is used to link two or more SPM beads through a specific analyte using the appropriate chemistries, as shown for model protein and nucleic acid systems in Fig. 1a,b. The formation of higher order bead structure induces changes in the optical and magnetic properties that have been used to quantify the presence and the concentration of a target molecule. The quantification of the beads’ response has been achieved using turbidimetry^18,19^, light scattering^20,21^, flow cytometry^2^ and reflectometry^16^. The isolation of the analyte carrying SPM beads could improve the performance of magnetic LOC assays by increasing the total number of beads that can be employed for analysis, facilitating the detection of low concentrations of analyte, and enabling the recovery of the target for further processing.

**Figure 2:**
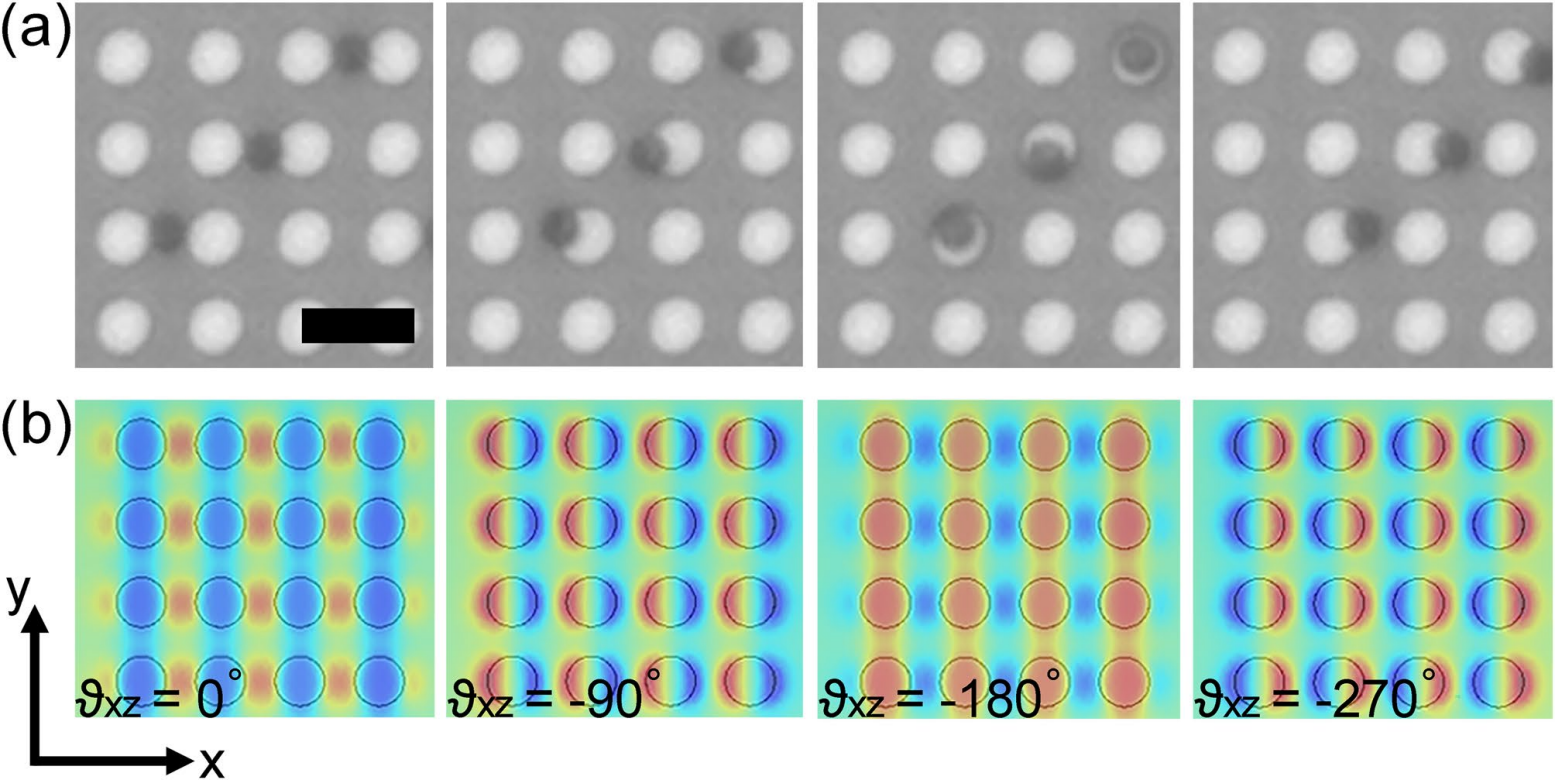
Transport of superparamagnetic beads on an array of circular micromagnets in an external magnetic field. (**a**) Optical microscope images of the motion of 2.8 μm SPM beads on the MMA, i.e., ϑ_xz_ increasees from 0° to − 270° across these images. The rotation of an external magnetic field in the *xz*-plane transports the SPM beads across the array of micromagnets in the *x*-direction. (**b**) FEMs of the electromagnetic field in the *xy*-plane shows the local magnetic flux density distribution for different orientations of the applied field. The warm colored areas correspond to the maxima of the magnetic flux density and the regions where the beads are most often found. The simulations were performed by imposing a micromagnet magnetization of 80 kA/m and an external field with a flux density of 30 G. Scale bar is 10 μm.

Precise control of the transport of SPM beads can be achieved through the combination of a rotating magnetic field with the microfabricated magnet arrays (MMA)^12,22–25^. Figure 2 presents a sequence of micrographs in which SPM beads are transported across an array of circular micromagnets (Fig. 2a) and the results of a finite element modeling (FEM) of the local magnetic flux density distribution on this MMA in the presence of an external magnetic field (Fig. 2b). The warm color regions correspond to the magnetic flux density maxima that move across the array following the rotation of the applied magnetic field (orientation of the field varied from ϑ_xz_ = 0° to − 270°). The magnetic force focuses the SPM beads to the point of maximum flux density and thus they travel across the array at a speed defined by the frequency of rotation of the external field. A simplified one-dimensional transport model has been used to describe the motion of SPM beads based on the magnetic force and hydrodynamic drag forces^26^. Significant improvements have been made to modelling the transport behavior of SPM beads on micromagnets by taking into account their hydrodynamic interaction with the substrate but are beyond the scope of this article^27–29^. When the rotational frequency of the external field (*ω*) is slow the beads are transported across the micromagnets at a constant velocity equal (*v*) in the ‘phase-locked’ regime $v = \omega \frac{{\text{d}}}{2\pi }$, where *d* is the center-to-center distance between adjacent micromagnets. Above a critical frequency, *ω*_*c*_, the beads begin to slip with respect to the translating magnetic flux density landscape due to the magnitude of the hydrodynamic force. The critical frequency has a dependence on the characteristic properties of the SPM beads and MMA.

$$ \omega_{c} = \frac{{\chi \mu_{0} \sigma_{0} (H_{ext} )}}{18\eta }(2\pi \beta )^{2} e^{ - 2\pi \beta } , $$

where *χ* is the beads’ magnetic susceptibility, *μ*_*o*_ is a constant denoting the magnetic permeability of vacuum, *σ*_*o*_ is an experimentally determined parameter that describes the field distribution generated by the micromagnets, *H*_*ext*_ is the magnetic field, *η* is the viscosity of the surrounding medium and *β* = *r/d* (where *r* is the radius of the bead). The slipping is observed as a rocking motion between adjacent micromagnets superimposed on the time-averaged linear velocity, which becomes zero at high frequencies. In the ‘phase slipping’ regime the velocity of the beads is approximately.

$$ v = \left[ {\omega - (\omega^{2} - \omega_{c}^{2} )^{1/2} } \right]\frac{d}{2\pi }. $$

The dependence of the critical frequency on the bead properties makes it possible to simultaneously separate bead populations due to differences in size, magnetization, or aggregation state, a technique we refer to as nonlinear magnetophoresis (NLM)^26,30,31^. NLM allows the transport of high densities of SPM beads, i.e., *ca.* 10^5^ micromagnets per cm^2^, with sub-µm precision without the consumption of carrier fluid. The absence of nonspecific magnetic field induced bead aggregation makes NLM well suited for LOC applications based on MBA assays.

This article builds on the recently published direct detection of < 200 copies of the *UL27* gene of herpes simplex virus (HSV) 1 using single particle tracking on a MMA using reflectometry^16^. We have evaluated four different shapes of micromagnets for SPM bead transport and separation, i.e., circular (C), square (S), triangular (T), and rectangular (R) micromagnets. The shapes and the overall configuration of a MMA tested in this study have been selected having in mind the capabilities of the photolithographic lift-off process used for fabricating the MMAs. It is understood that other shapes of micromagnets may be envisaged, in accordance with the limitations of the fabrication method of choice. The results described herein can be nonetheless generalized and taken into consideration for the design of micromagnets having a shape other than those described in this work. The transport behavior of SPM beads on the MMAs have been linked to the micromagnets' lateral dimension and shape, which define the spatial distribution of the local magnetic flux density. We also demonstrate that the local magnetic flux density increases with the overall size of the micromagnets. These factors are linked to the observed critical frequency of the beads on the different arrays and importantly to the transport behavior of single beads and bead aggregates. We apply these MMAs to NLM separation of MBA assay products for the detection of a model protein biomarker and single-stranded DNA (ssDNA), as illustrated in Fig. 1c. Conventionally, nucleic acid testing (NAT) is based on polymerase chain reaction (PCR), which amplifies the most common sequence using significant volumes of reagents and complex and time-consuming procedures^32–37^. A direct, rapid and highly sensitive alternative to PCR methods, such as, single particle tracking using MMA, could allow NAT to be easily applied at emerging applications, i.e., next-generation sequencing and epigenetics, as well as POCT. The results presented here provide useful guidelines for the design of NLM separation systems with the aim of improving on-chip detection.

## Results and discussion

### NLM transportation and separation optimization

**Figure 3:**
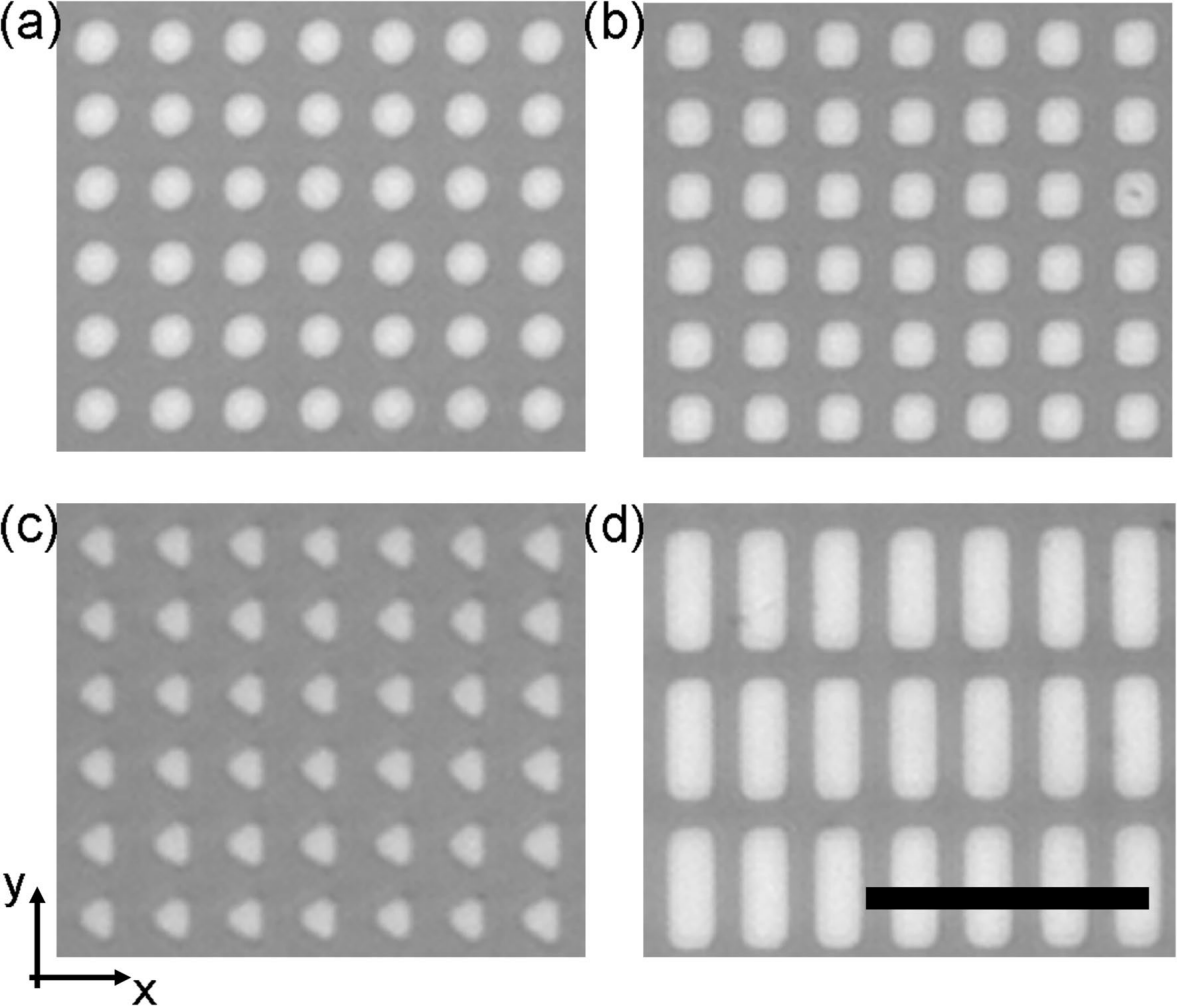
Optical microscope images of the four micromagnet arrays. (**a**) Circular micromagnets with 5 μm diameter and 8 μm centre-to-centre distance. (**b**) Square micromagnets with 5 μm lateral dimension and 8 μm centre-to-centre distance. (**c**) Triangular micromagnets with 5 μm lateral dimension and 8 μm centre-to-centre distance. (**d**) Rectangular micromagnets with 5 × 12.5 μm lateral dimensions and centre-to-centre distance 8 μm in the x-direction and 16 μm in the y-direction. Scale bar is 30 μm.

Figure 3 presents the four MMAs designed and fabricated for this study, consisting of rectilinear arrays of micromagnets of specific shape with a critical dimension of approximately 5 µm that are separated by a 3 μm gap. The NLM transport behavior of each MMA was characterized by measuring the velocity of 2.8 μm SPM streptavidin beads and ‘dimers’, which were formed by the reaction of these beads with biotinylated bovine serum albumin (BBSA), for increasing rotational frequencies of the applied magnetic field. Dimers were considered rather than higher order aggregates as they are the most common SPM bead assembly observed in the MBA assays and their transport properties are well defined. Four criteria were used to evaluate the relative performances of the micromagnet designs, i.e., critical frequency (*ω*_*c*_), immobilization frequency (*ω*_*i*_), relative velocities of the beads at *ω* > *ω*_*c*_, and their trajectories across an MMA. For the first criterion, a high *ω*_*c*_ is desirable as it allowed rapid separation to be achieved, which reduces the overall processing time of the assay. The second criterion, the *ω*_*i*_ is related to the separation efficiency of the NLM technique for different beads assemblies. For operational purposes the immobilization frequency has been defined as the frequency at which the average bead velocity decreased to less than 10% of its maximum value^17,18^. It has been observed that large differences in *ω*_*c*_ and *ω*_*i*_ correspond to a loss in separation efficiency due to the imprecise control of the frequency at which the single beads and dimers are separated^18^. The third criterion is related to the relative velocity of the different bead assemblies at *ω* > *ω*_*c*_. NLM separation efficacy is optimized when this difference is maximized. Finally, efficient separation results from controlled transport behavior but shifts in the orientation of the beads were observed from one row of micromagnets to another in several of the geometries.

**Figure 4:**
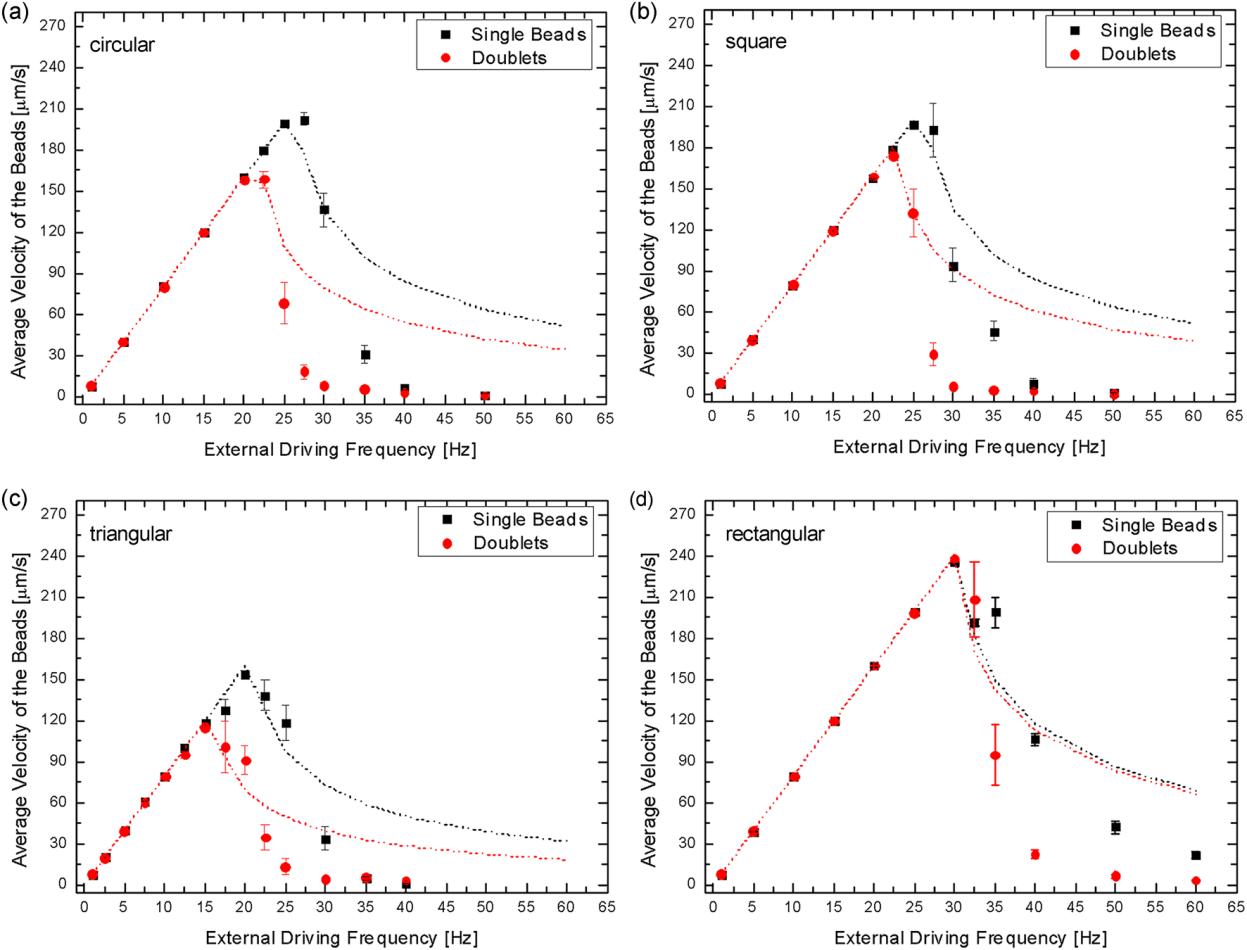
Magnetophoretic velocity profiles of single beads and dimers as a function of frequency of rotation of the external magnetic field. (**a**) Velocity profile on circular magnets. The estimated *ω*_*c*_ for single beads was ~ 27 Hz, whereas for the dimers it was ~ 22.5 Hz. (**b**) Velocity profile on square magnets. The estimated *ω*_*c*_ for single beads was ~ 27 Hz, whereas for the dimers it was ~ 23.5 Hz. (**c**) Velocity profile on triangular magnets. The estimated *ω*_*c*_ for single beads was ~ 21.5 Hz, whereas for the dimers it was ~ 16.5 Hz. (**d**) Velocity profile on rectangular magnets. The estimated *ω*_*c*_ for single beads was ~ 31 Hz, whereas for the dimers it was ~ 30.5 Hz. The theoretically predicted profiles have been plotted in the graphs as dotted lines. The error bars represent the standard error of the mean.

Figure 4 presents the velocity profiles of single SPM beads and dimers on the four MMAs as the frequency of the external magnetic field was increased. Each velocity measurement in Fig. 4a–d is the result of approximately 15 microscopic measurements on a single bead or dimer on the circular, square, triangular, and rectangular MMA, respectively. At low frequencies the velocity of the beads was not dependent on their aggregation state, as expected from the local magnetic flux densities, but above *ω*_*c*_ the velocities started decreasing until *ω*_*i*_ was reached. The SPM beads and dimers had significantly different transport properties at *ω* > *ω*_*c*_ on the four MMAs. The dimers showed a lower *ω*_*c*_ than single SPM beads on C, S and T-micromagnets, whereas they had a critical frequency similar to the single beads on R. T-micromagnets had the lowest critical frequencies, i.e., 21.5 Hz for single beads and 16.5 Hz for dimers. R-micromagnets showed the highest critical frequencies, i.e., 31.0 Hz for single beads and 30.5 Hz for dimers. C and S-micromagnets showed an intermediate value of critical frequency for single beads, i.e., 27.0 Hz, and the dimer critical frequency decreased to 22.5 Hz and 23.5 Hz, respectively. The difference in the critical frequencies of the beads in the four arrays is attributed to the difference in the local magnetic flux density generated by the four micromagnet shapes, which is discussed in detail below. The variations in the lateral dimension of the micromagnets, and consequently in their volume, led to lower (or higher) magnetic force and therefore to lower (or higher) critical frequency.

Significant differences were also observed in the immobilization frequencies of SPM beads and aggregates on the four MMAs, as observed in Fig. 4. The dimers had a *ω*_*i*_ of 25 Hz for T, 30 Hz for C and S, and 40 Hz for R-micromagnets, whereas the *ω*_*i*_ of single beads was 25 Hz for T, 40 Hz for C and S, and 60 Hz for R-micromagnets. To facilitate the analysis of the sharpness of the transition from “mobile” to “immobile” behavior we introduce the parameter Ω = *ω*_*c*_*/ω*_*i*_. The parameter Ω for single beads was 0.61 for T, 0.67 for C and S, and 0.51 for R-micromagnets. The parameter Ω for the dimers was 0.66 for T, 0.75 and 0.78 for C and S, respectively, and 0.76 for R-micromagnets. The higher values of Ω for dimers (with respect to single beads) is associated with the tendency of the dimers to change their orientation of motion when the driving frequency increased to values higher than *ω*_*c*_, i.e., it was found that the longitudinal axis of the dimers (defined as the axis between the two SPM beads) rotated from normal to parallel to the direction of motion at high *ω*. This led to a reduction of the total magnetic force experienced by the dimers and thus to a reduction in the immobilization frequency. Finally, the bead motion was observed to be stable across each of the MMAs except for R-micromagnets, where a shift of the beads’ trajectory was occasionally observed in the *y*-direction, leading to variations in their apparent x-direction velocities.

**Figure 5:**
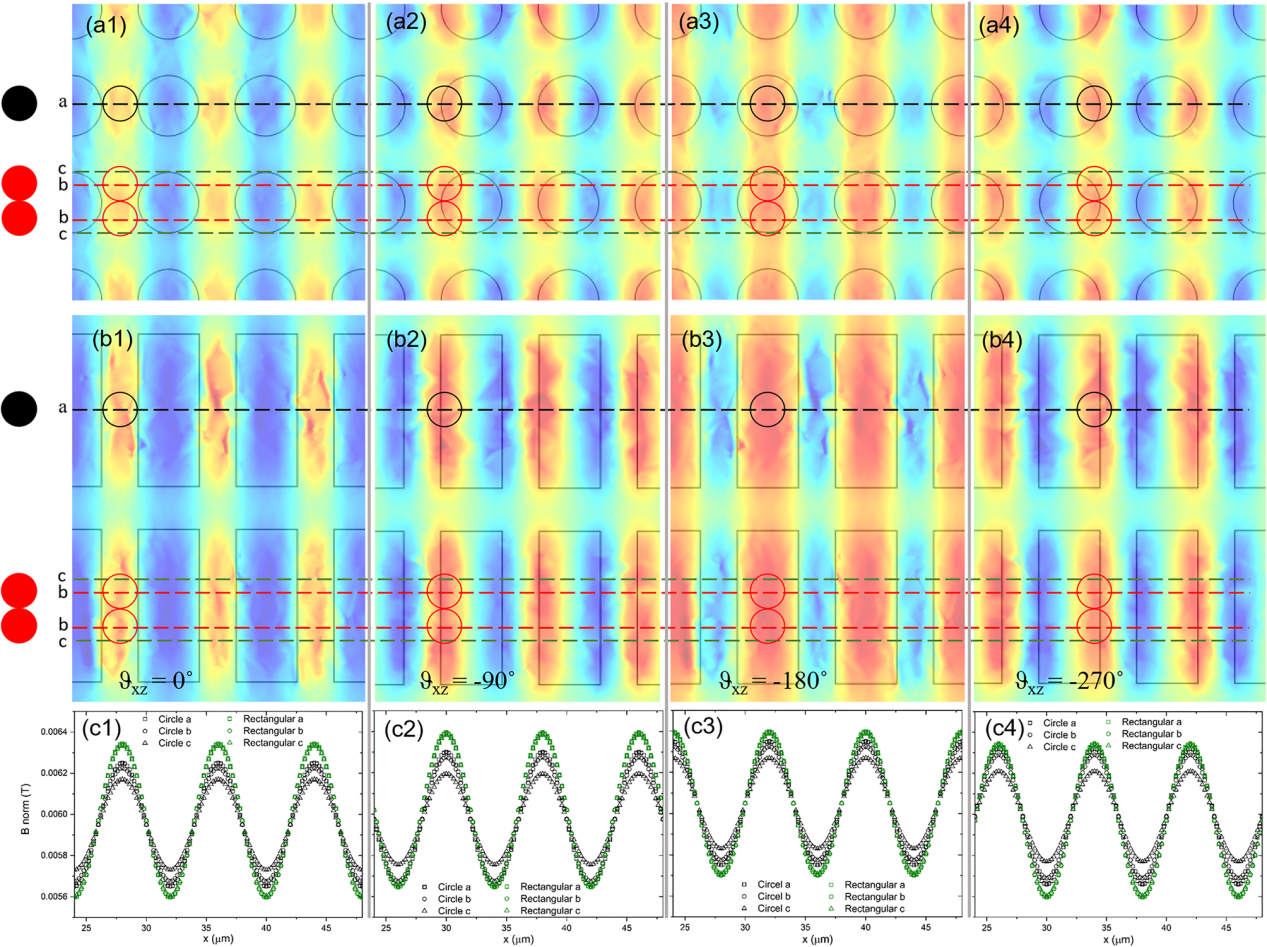
Analysis of the magnetic flux density and transport behavior on the circular and rectangular MMAs. (**a**) Results of FEM of the magnetic flux density 1.4 µm above the spin-on glass surface on a circular micromagnet array as the external magnetic field is rotated through four positions. The position of the single beads and dimers have been drawn to scale on the FEM results at the point of maximum flux density. The dash lines “*a*” are located in the center of the trajectory of a single bead in *y* direction; the dash lines “*b*” are located in the center of the trajectory of the dimers; and the dash lines “*c*” are 1.1 μm offset in y-direction from dash lines “*b*”. The FEM were performed using a micromagnet magnetization of 80 kA/m and an external field with a flux density of 30 G. (**b**) Results of FEM of the magnetic flux density 1.4 μm above the spin-on glass surface on a rectangular micromagnet array as the external magnetic field is rotation through four positions. (**c**) Normalized magnetic flux density along the *a*, *b* and *c* lines for the circular and rectangular micromagnet arrays.

The transport properties of single beads and dimers were closely related to the magnetic flux density on the micromagnetic array and to their lateral dimensions. Previous studies have revealed that SPM beads traveling on C-micromagnets move to the point of maximum flux density created by the micromagnet and external magnetic field, as shown in Fig. 2^26^. Figure 5 presents the results of finite element modeling (FEM) of the local magnetic flux density distribution in the plane of the SPM beads for the C and R for different orientations of the magnetic field (i.e., the figures labeled 1 have a *θ*_*xz*_ = 0°, label 2 *θ*_*xz*_ = − 90°, label 3 *θ*_*xz*_ = − 180° and label 4 *θ*_*xz*_ = − 270°). The SPM beads and dimers have been drawn to scale on Fig. 5a,b at the point of maximum magnet flux density. The single beads are smaller than the C-micromagnet, as shown in Fig. 5a, while approximately 6% of the volume of the dimers fell outside for a C-micromagnet. The magnetic flux density on R, shown in Fig. 5b, suggest that the single beads and dimers experienced similar magnitudes of magnetic flux density for all orientations of the external magnetic field. Figure 5c presents the magnitude magnetic flux density along the center of a single bead, center of the dimers and edges of the dimer for C and R-micromagnets as a function of x-direction. These results confirm that the magnetic flux density distribution on R micromagnets for a single beads and dimer is substantially the same. It is also clear that the magnitude of the magnetic flux density for the single beads on C was 62.5–75% smaller than on R-micromagnets. The change in magnitude of the magnetic field densities is in qualitative agreement with the change in transport properties, i.e., *ω*_*c,C*_ is 87% of *ω*_*c,R*_ for the single beads. The magnitude of the change in the magnetic flux density was also observed to be significantly lower along the outer edges for C-micromagnets, which suggests that the *ω*_*c*_ for the dimers should be lower than single beads. Again, this was consistent with the experimentally determined transport properties of the dimers, i.e., *ω*_*c,C*_ is 77% of *ω*_*c,R*_ for the dimers.

Figure S1 presents the FEM results for the magnetic flux density distributed for S and T-micromagnets at the four orientations of the external magnetic field (i.e., the figures labeled 1 have a *θ*_*xz*_ = 0°, label 2 *θ*_*xz*_ = − 90°, label 3 *θ*_*xz*_ = − 180° and label 4 *θ*_*xz*_ = − 270°). The magnetic flux density profiles experienced by single beads and dimers on S and T are presented in Figure S1f. Obviously, the magnitude of the magnetic field density maxima for the single beads and dimers was smaller for T than the C-micromagnets. This is consistent with the observed decrease in *ω*_*c*_ for the single beads and dimers on T, i.e., *ω*_*c,T*_ is 80% of *ω*_*c,S*_ for the single beads and *ω*_*c,T*_ is 68% of *ω*_*c,S*_ for the dimers. Two other trends are clear from Figure S1. First, the change in the magnitude of the magnetic flux density for single beads on S was similar in magnitude to C-micromagnets for *θ*_*xz*_ = 0 and − 180° but was significantly larger for *θ*_*xz*_ = − 90 and − 270°. The change in the magnitude of the magnetic flux density was also observed to be significantly smaller along the outer edges on the S-micromagnets. This suggests that the *ω*_*c*_ for the dimers should be lower than single beads, which was consistent with the observed transport properties. Second, the shape of the magnetic flux density profiles for T are not symmetric. This suggests that these arrays will have asymmetric NLM separation behavior based on the x-direction of motion of the SPM beads.

The NLM microscope experiments allowed us to characterize the transport properties of different MMAs, in terms of critical frequency, stability of the motion, and separation capabilities. The magnetic flux density on all four MMAs forced the dimers into an orientation in which they traveled with their longitudinal axis orthogonal to the direction of motion for *ω* < *ω*_*c*_. The lateral dimension of the dimers exceeded the lateral dimension of the C, S and T-micromagnets. Thus, the dimer experienced a lower local magnetic potential energy on these MMAs compared to single beads, resulting in lower critical frequencies^30^. In the case of R-micromagnets, the magnetic flux density experienced by the dimers was comparable to that experienced by the single beads due to the shape of the magnetic flux density maxima. The results highlighted the sensitivity of the NLM technique to the local magnetic flux density and confirmed the versatility of the MMA design, which obviously can be adjusted to meet specific experimental requirements associated with SPM beads and their aggregate properties. Based on the criteria previously defined it was concluded that for the applications of interest of this work the C and S-micromagnets were the most suitable designs, i.e., these two shapes gave the highest critical frequency combined with the minimum overlap between the velocity profiles of the single bead and dimer populations. R was less desirable due to the unpredictable transport behavior of the SPM beads and limited capacity to distinguish between single beads and dimers.

### On-chip detection of the *UL27* gene from HSV-1 using an MBA assay

We tested an S-micromagnets chip to determine its suitability for the separation and detection of an MBA assay based on the separation of the velocity profiles of the single SPM beads and dimers. Two biological targets were employed in these experiments, i.e., initial characterization was performed using BBSA as the analyte and subsequent experiments were performed with the *UL27* gene of HSV-1. In the MBA assay, the SPM beads were first incubated with the analyte for approximately 5 min and then a magnetic field was used to induce rapid bead-bead reaction and separation (see “Methods” section for details). This reaction product was introduced to the S-chip and ingle beads were separated from the aggregates by applying an external magnetic field. At the same time, the percentage of single beads and aggregates in the samples was evaluated microscopically, i.e., 100 beads were counted for each target concentration.

**Figure 6:**
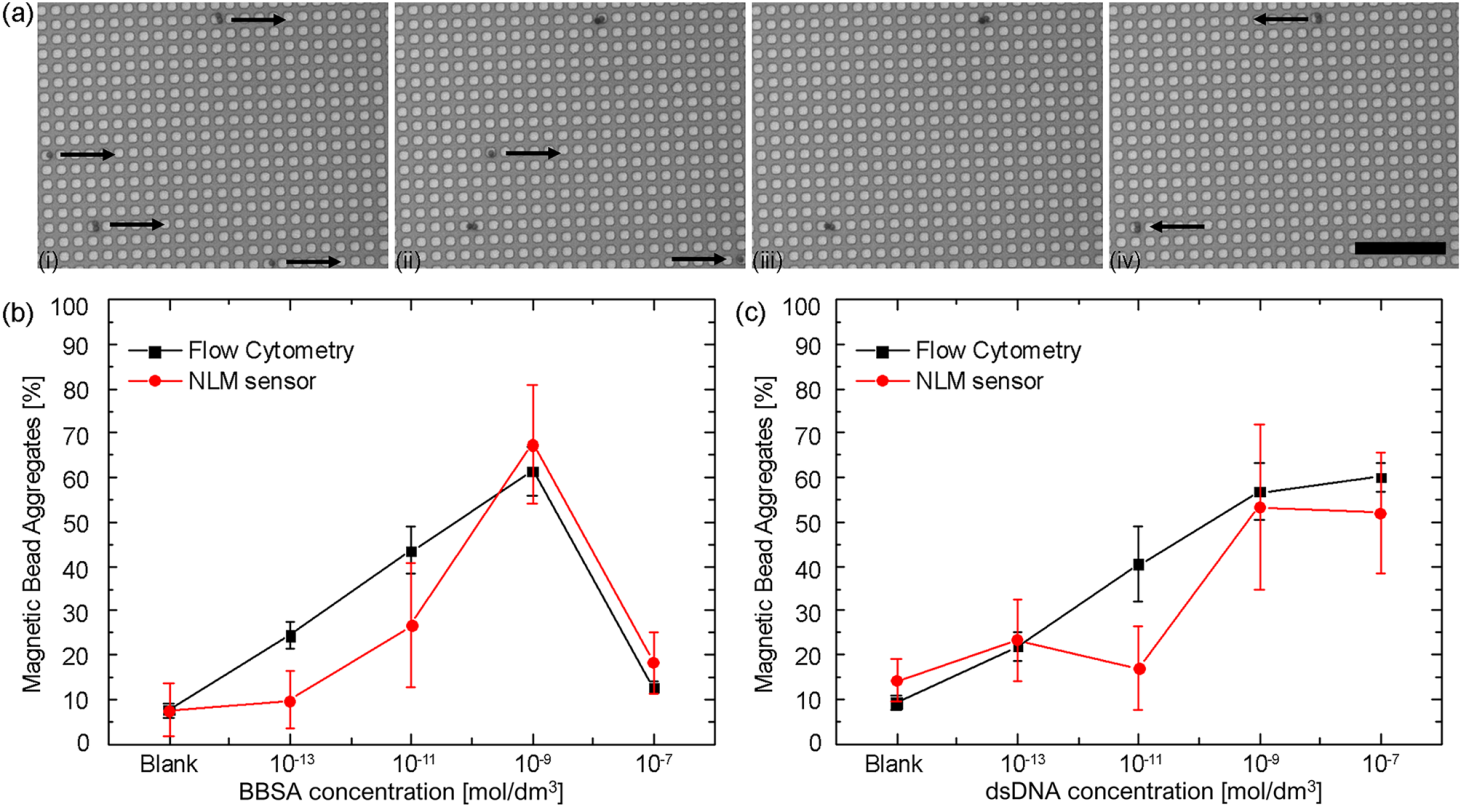
NLM and flow cytometry analysis of the MBA assays for biotinylated bovine serum albumin and double-stranded DNA. (**a**) Optical micrographs of the separation process. (i) Single beads and dimers travel on the array of S-micromagnets at low driving frequency, (ii,iii) when the driving frequency is increased, the average velocity of the dimers decreases, making it possible to separate them from the single beads, and (iv) the sense of rotation of the applied field is inverted and the dimers are transported in the opposite direction with respect to single beads. (**b**) Percentage of aggregates in the samples for increasing BBSA concentration, measured on the NLM chip and with a flow cytometer. (**c**) Percentage of aggregates in the samples for increasing dsDNA concentration, measured on the NLM chip and with a flow cytometer. Scale bar is 50 μm.

Figure 6a presents a sequence of microscope images showing the NLM separation process. Initially, all the beads were transported to the right at *ω* < *ω*_*c*_, as shown in Fig. 6a,i. The driving frequency was increased to approximately 30 Hz where the average velocity of the dimers decreased, enabling their separation from the monomers, as shown in Fig. 6aii,iii. The magnetic field was maintained at approximately 30 Hz for several seconds, thus allowing the monomers to travel off the right edge of the micromagnet array. The orientation of rotation of the external applied magnetic field was then reversed, leaving the aggregates separated from the monomers, as shown in Fig. 6aiv. Separation efficiency was defined as ε = 1 − f_sb_ − f_agg_, where *f*_*sb*_ is the fraction of single beads that were immobilized on the array and *f*_*agg*_ is the fraction of the dimers that were lost, which is attributed to their NLM transport off the array. The average efficiency for NLM separation of BBSA aggregates was *ε* = 0.93 ± 0.02 over the range of concentrations tested in this study, i.e., from 10^–13^ to 10^–7^ mol dm^−3^ BBSA. The separation efficiency was not found to be dependent on the target concentration but appears to be primarily limited by the nonspecific adhesion of single beads with the surface of the array or the interaction of single beads. The average efficiency for the separation of the HSV-1 *UL27* gene aggregates was *ε* = 0.89 ± 0.07 over the range of tested concentrations. The lower efficiency and the higher standard deviation in this case were linked to the nonspecific interaction of the dimers with the NLM chip, which may be due to the well-known interaction of DNA with silica surfaces, reducing the efficiency of the process.

The MBA reaction products were analyzed to determine the fraction of dimers and single beads in the samples. Figure 6b presents the NLM (red curve) and flow cytometer (black curve) results of the MBA assay for BBSA in which the fraction of aggregated beads is observed to be a nonlinear function of concentration. The percentage of aggregates increased from 18.3% ± 6.9% to 67.4% ± 13.4% as the BBSA concentration decreased from 10^–7^ to 10^–9^ mol dm^−3^, respectively. It decreased to 26.7% ± 14.0% for a concentration of 10^–11^ mol dm^−3^ and to 9.9% ± 6.3% for a concentration 10^–11^ mol dm^−3^. The lower percentage of aggregates for high BBSA concentration was due to the saturation of the streptavidin sites on the magnetic beads, which is the so-called hook effect. Figure 6c shows the results for the *UL27* gene MBA assay. For a DNA concentration of 10^–7^ mol dm^−3^ the percentage of aggregates was 52.0% ± 13.4%. The result was similar for a DNA concentration of 10^–9^ mol dm^−3^ for which the percentage of aggregates was 53.4% ± 18.4%. For lower concentration, 10^–11^ mol dm^−3^ and 10^–13^ mol dm^−3^, the percentage of aggregates decreased to 17.0% ± 9.2% and 23.4% ± 9.3%, respectively.

The dose–response curves for the MBA *UL27* assay exhibit a nonlinear behavior. We observed approximately a threefold difference in the signal over a target concentration range of four orders of magnitude (from 10^–13^ to 10^–9^). In previous publications on bead-based assays related to GMR detection^38^, or to fluidic force discrimination^39^, the sub-linearity of the dose–response curves at high target concentration was attributed to magnetic and steric hindrance effects. At low target concentrations, the formation of SPM bead dimers is statistically favored over the formation of larger bead clusters^21^. At higher target concentrations each SPM bead captures multiple target molecules, and the formation of bead aggregates formed by multiple SPM beads tends to occur (confirmed by microscopy observation), a phenomenon that may decrease the efficiency of the assay due to the lower binding capabilities of bead clusters^40^. SPM beads with smaller size (i.e., in the hundreds of nm range) could be used to improve the sensitivity of the assay, however the characteristic dimensions of the micromagnet arrays would need to be adjusted accordingly, in order to maintain efficient transport and separation capabilities: the lateral dimension and the gap between adjacent micromagnets should be reduced for achieving smooth transport of such small beads.

These NLM results are in qualitative agreement with the flow cytometry measurements presented in Fig. 6b,c. The difference in the results of the MBA assay between the flow cytometer and NLM were attributed to several factors. The primary difference appears to be associated with non-specific bead-surface and bead-bead interactions in the NLM will lead to an undercounting of the bead aggregates, i.e., only the bead aggregates that were mobile were counted in the NLM assay. The second difference is associated with the total number of beads that are analyzed. The flow cytometer detects thousands of events per second over a 10–100 s period, while in our NLM we only counted several hundred beads. The limitations of the NLM system can be reduced by integrating non-stick coatings and implementing a detection scheme that is able to count more beads in an automated fashion.

## Conclusions and outlook

In this article, we examined the NLM transport of SPM beads based on different shape and size micromagnets. It was found that the transport behavior of both the SPM bead monomers and dimer was closely related to magnetic flux density produced by the micromagnet array. For the monomers, the transition from linear to nonlinear transport, i.e., the magnitude of *ω*_*c*_ and *ω*_*i*_, was observed to increase in frequency with the increase in the maximum magnetic flux density, e.g., see Fig. 5c1–4 and Figure S1f1–4. This is consistent with our understanding that the magnetic force applied to the superparamagnetic bead is proportional to the gradient of the magnetic field at the position of the bead, which is at the magnetic field maximum in the linear transport regime. For SPM bead dimers, the transition from linear to nonlinear transport was found to be closely related to the shape and size of the micromagnets relative to the dimers. For example, the R-micromagnet array produced high local magnetic flux densities and resulted in a higher frequency *ω*_*c*_ and *ω*_*i*_, as shown in Fig. 4d. However, the transport behavior of the monomers and dimers on the R-micromagnets was substantially indistinguishable. This can be understood to result from the fact that the monomer and dimer experience similar magnetic flux density gradients through their entire volume. The C, S and T arrays produced lower local magnetic flux densities and were observed to have lower frequency *ω*_*c*_’s and *ω*_*i*_’s. However, these MMAs were able to separate monomers from dimers due to the significant difference in the magnetic flux densities at the edges of the micromagnet, which disproportionally influenced the transport properties of the dimers. These results suggest that high-efficiency NLM separation will be achieved with micromagnets that have lateral dimensions that are larger than monomers but smaller than dimers. This conclusion is subject to the observation that dimers can change their orientation at high NLM frequencies to reduce the hydrodynamic force.

An S-chip was used to separate and detect two different biological targets to evaluate the efficiency of the NLM separation process and detection of dimers for the MBA assay using a simple optical microscope. Our previous studies have demonstrated that sensitivity of this technique is highly dependent on the parameters used for the MBA assay, i.e., type of beads used, surface chemistry, reaction times and volumes. After the SPM beads are added to the MMA the desired biological targets were isolated from the sample in a few seconds after the rotating magnetic field was activated. The sensitivity of the NLM MBA assays was similar to those obtained from a flow cytometer, with both techniques analyzing as many as 10^5^ single beads and dimers. The lower aggregation level measured with NLM appears to result from the adhesion of the SPM dimers and larger aggregates to the silicon dioxide MMA surface, resulting in decreases in the apparent number of aggregates. We anticipate that the sensitivity of the NLM MBA assays will be equivalent to the flow cytometer once the nonspecific interactions of the SPM beads has been mitigated using a passivating surface chemistry. NLM also allowed us to work with very small number of bead aggregates, and thus molecules, which could have significant advantages for next-generation sequencing and epigenetic analyses where it is desirable to avoid PCR amplification.

A microscope was used to detect the SPM bead aggregates in this study, which was useful for the characterization of the transport processes and had single particle sensitivity, but obviously is not easily automated. Advances in optical and magnetic sensor design should allow detectors to be integrated into MMAs and the resulting LOC device promised to allow rapid, high-sensitivity analysis of nucleic acids, proteins, viruses or cells to be performed from complex media with minimal consumption of fluids.

## Methods

### Analysis of NLM transport on the MMAs

The NLM system used in this study has been described in previous publications^30,31^. Briefly, the applied magnetic field rotating in the *xz*-plane was generated by three iron core electromagnets supplied with sinusoidal signals with a 90° phase difference via a two-channel function generator (Tektronix, Beaverton, Oregon, USA). This signal was amplified to the desired current using two programmable amplifiers (Kepco, Flushing, NY, USA). An ellipsoidal rotating field, with components of 30 G in the x-direction and 35 G in the z-direction, was used consistently in all the experiments. The transport of the SPM beads in the device was characterized with an Axioskop2 optical epi-illumination microscope (Zeiss, Oberkochen, Germany).

The MMAs presented in Fig. 2 were tested with commercially available 2.8 μm (3% CV) streptavidin coated beads (Invitrogen, Carlsbad, CA). These beads have a saturation magnetization of 11.2 A m^2^ kg^−1^ and density of 1.6 × 10^3^ kg/m^3^. The bead aggregates were formed by BBSA at a concentration 10^–9^ mol dm^−3^. The experimental data reported in this paper refer to single beads and to aggregates formed by two beads, which was the most statistically favored shape in the samples at this BBSA concentration. The rare clumps of beads were not considered in these measurements since their behavior was not predictable, mainly due to an out-of-plane rotation during their in-plane motion across the MMA. The rotational frequency of the magnetic field was varied from 1 Hz up to 60 Hz, with increasing frequency steps. The distance travelled by the beads over a defined number of microscope frames was measured manually in the Zeiss AxioVision software. On average 15 beads or aggregates were analysed for each data point presented. In the high frequency regime, the SPM beads rapidly oscillated without moving from one micromagnet to the next one. In this regime the beads were classified as “immobile”. The ω_c_ of the beads was defined as the frequency at which the average bead velocity started to diverge from the velocity of the translating potential energy landscape, ω_d_/2π, and was estimated based on the bead frequency response curves.

### MMA design and fabrication

The micromagnet fabrication followed a standard photolithographic lift-off process. The micromagnets were fabricated on a silicon wafer via electron-beam deposition using 10 nm chromium, 100 nm cobalt, and 10 nm chromium. The bottom layer of chromium was used to enhance the adhesion of the micromagnets to the silicon substrate, whereas the top layer of chromium protects the cobalt from oxidation. A 600 nm thick spin-on-glass (Filmtronics, Butler, PA) layer was added on-top of the micromagnets to create a planar surface and ensure further protective layer for the cobalt. After the fabrication process the micromagnets were magnetized along the positive x-direction with a 11 kg impulse magnetizer (ASC Scientific, Carslbad, CA). Four different shaped micromagnets were fabricated, i.e., circular (Fig. 3a, 5 μm diameter), square (Fig. 3b, 5 μm side), equilateral triangle (Fig. 3c, 5 μm side), and rectangular (Fig. 3d, 5 × 12.5 μm sides). The centre-to-centre distance between adjacent magnets in the *x*- and *y*-direction was 8 μm for circular, triangular and square micromagnets, whereas it was 8 μm in the x-direction and 16 μm in the *y*-direction for rectangular micromagnets.

### MBA assay for biotinylated bovine serum albumin and the *UL27* gene of Herpes simplex virus-1

Figure 1 presents a schematic of magnetic bead aggregation (MBA) assays in which BBSA and DNA react with functionalized SPM beads leading to the formation of dimers and aggregates of higher order. Figure 1a illustrates the MBA assay used to detect the model protein BBSA based on the aggregation of streptavidin coated SPM beads (Invitrogen). The BBSA (Thermofisher Scientific) with nominally 7 biotins per albumin molecules was used as received and diluted in defined amounts in phosphate buffer saline (137 mM NaCl, 2.7 mM KCl, 10 mM Na_2_HPO_4_, 1.8 mM KH_2_PO_4_), pH 7.4, with 0.1% Tween (PBST). The assay was performed by adding streptavidin SPM beads to 300 μl of the BBSA solutions to achieve a final bead concentration of 10^7^ mL^−1^. The solution was incubated at room temperature for 5 min and then subjected to magnetic field activated aggregation, i.e., the sample was placed in a 2.5 kg magnetic field for ~ 1 min at which time the majority of the beads came out of solution. The tube was then rotated 90° in the magnetic field to allow the SPM beads to roll over each other and react. Approximately 100 μl samples of the product of 10^−7^, 10^−9^, 10^−11^ and 10^–13^ mol L^−1^ BBSA reactions were added to a S-micromagnet MMA for separation and detection with an optical microscope.

Figure 1b shows the MBA assay performed on double-stranded DNA (dsDNA). In this study two 25mer DNA probes were hybridized with a 75-mer single-stranded DNA (ssDNA) from herpes simplex virus-1 (HSV-1). The biotin–HSV–1P1 probe was modified with biotin on 3′ end and the Dig–HSV–1P1 probe was 5′ labelled with digoxigenin (sequences presented in Table S1). These DNA probes were complementary to different ends of the HSV-1 KOS gB-75mer ssDNA. The dsDNA was prepared by mixing the two probes at 1 nmol concentrations with the 75mer ssDNA at a 1 nmol concentration in a volume of 100 µl annealing buffer (10 mM TrisHCl, 1 mM EDTA, 100 mM NaCl, pH 7.5) for 10 min at 85 °C. After hybridization the dsDNA samples were stored in annealing buffer at 4 °C. The antidigoxigenin SPMs were prepared by reacting 30 mg biotinylated antibody (Abcam, Cambridge, England) with the streptavidin beads (1 mg) in 1 mL PBS buffer for 30 min on a rotating wheel at room temperature. The antibody coated beads were then washed with PBST buffer and incubated with 0.05% biotin in 1 mL PBST buffer for 30 min at room temperature. After a washing step with PBST buffer, the beads were stored at 4 °C in 1 mL PBST buffer containing 0.1% BSA. Samples of dsDNA were prepared by dilution at concentrations of 10^–7^, 10^–9^, 10^–11^ and 10^–13^ mol dm^-3^ of TPT buffer (5 mM Tris–HCl, 0.5 mM EDTA, 5 mM phosphate buffer, 1 M NaCl, 0.05% Tween 20, pH 7.5). Streptavidin beads were added to the solution containing the target dsDNA, and the mixture was incubated at room temperature for 30 min. After this step, the dsDNA coated beads were washed with TPT buffer and reacted with biotin (0.5%) in 1 mL of TPT buffer for 30 min. After washing with TPT buffer, the beads were incubated with antidigoxigenin functionalized SPM beads for 30 min on a rotating wheel. The sample containing SPM beads was placed in a magnetic field of 2.5 kg until most of the beads came out of solution. Once the SPMs were collected the tube was rotated by 90° in the magnetic field. Approximately 100 µl samples of the product were added to a S-micromagnet MMA for separation and detection with an optical microscope.

### Flow cytometry analysis of MBA products

Beads aggregation were also evaluated using a flow cytometer (Accuri C6, BD biosciences, UK) with two light scattering detectors and four fluorescence detectors. The percentage of monomers and aggregates in the sample were determined by gating individual areas of the scatter plots with the foreword scattering area (FSC-A) against the foreword scattering height (FSC-H). Individual monomers have lower FSC-A and FSC-H values, and aggregated beads are easily identified and discriminated from monomers due to the higher FSC-A and FSC-H values. Each assay was run in triplicate and the average values are plotted in all graphs.

## Supplementary Information

Supplementary Information.
