# Supplementary material for: Design of micromagnetic arrays for on-chip separation of superparamagnetic bead aggregates and detection of a model protein and double-stranded DNA analytes
Source: Sci Rep. 2021 Mar 5;11:5302. doi: 10.1038/s41598-021-84395-3 (PMC7935980; doi:10.1038/s41598-021-84395-3)

**Supplementary Information**

**Design of micromagnetic arrays for on-chip separation of superparamagnetic bead aggregates and detection of a model protein and double-stranded DNA analytes**

Stefano Rampini^1^, Peng Li^1^, Dhruv Gandhi^1^, Marina Mutas^1^, Ying Fen Ran^1^, Michael Carr^3,4^ Gil U. Lee^1,2^*

^1^ School of Chemistry, University College Dublin, Belfield, Dublin4, Ireland

^2^ Conway Institute for Biomolecular and Biomedical Research, University College Dublin

^3^ UCD National Virus Reference Laboratory, University College Dublin, Belfield, Dublin, Ireland

^4^ Global Institution for Collaborative Research and Education (GI-CoRE), Hokkaido University, Kita-ku, Japan

*Corresponding author: gil.lee@ucd.ie

**Table S1.** **Oligonucleotides probes used in this study.** The melting temperature, T_m,_ was based on theoretical calculations.

| **Notation** | **Sequence (5’→ 3’)** | **T_m_ (˚C)** |
| --- | --- | --- |
| Dig-HSV-1P1 | Digoxigenin -GCCGCGTTGCCATCGCGTGGTGCGA | 69.5 |
| Biotin-HSV-1P1 | TGGAACGAGGCCCGCAAGCTGAACC-Biotin | 64.7 |
| HSV-1 KOS gB- | GGTTCAGCTTGCGGGCCTCGTTCCACAGGGTCAGCTCGTGATTCTGTAGCTCGCACCACGCGATGGCAACGCGGC | 83.0 |

**Figure S1. Analysis of the magnetic flux density and transport behavior on the square and triangular MMAs.** (d) Results of FEM of the magnetic flux density 1.4 um above the spin-on glass surface on a square micromagnet array (large black squares) as the external magnetic field is rotated through four positions, i.e., d1, d2, d3 and d4 correspond to *θ_xz_* decreases from 0, -90, -180 and -270˚ across. The position of the single beads (small black circles) and dimers (small red circles) have been drawn to scale on the FEM results at the point of maximum flux density. The dash lines “*a”* are located in the center of the trajectory of a single bead in *y* direction; the dash lines “*b*” are located in the center of the trajectory of the dimers; and the dash lines *“c”* are 1.1 μm offset in y-direction from dash lines “*b*”. (e) Results of FEM of the magnetic flux density 1.4 um above the spin-on glass surface on a triangular micromagnet array (large black triangles) as the external magnetic field is rotated through four positions, i.e., e1, e2, e3 and e4 correspond to *θ_xz_* decreases from 0, -90, -180 and -270˚ across. (f) Normalized magnetic flux density along the *a*, *b* and *c* lines for the circular and rectangular micromagnet arrays as the external magnetic field is rotated through four positions, i.e., f1, f2, f3 and f4 correspond to *θ_xz_* decreases from 0, -90, -180 and -270˚ across. The FEM were performed using a micromagnet magnetization of 80 kA/m and an external field with a flux density of 30 G.

.
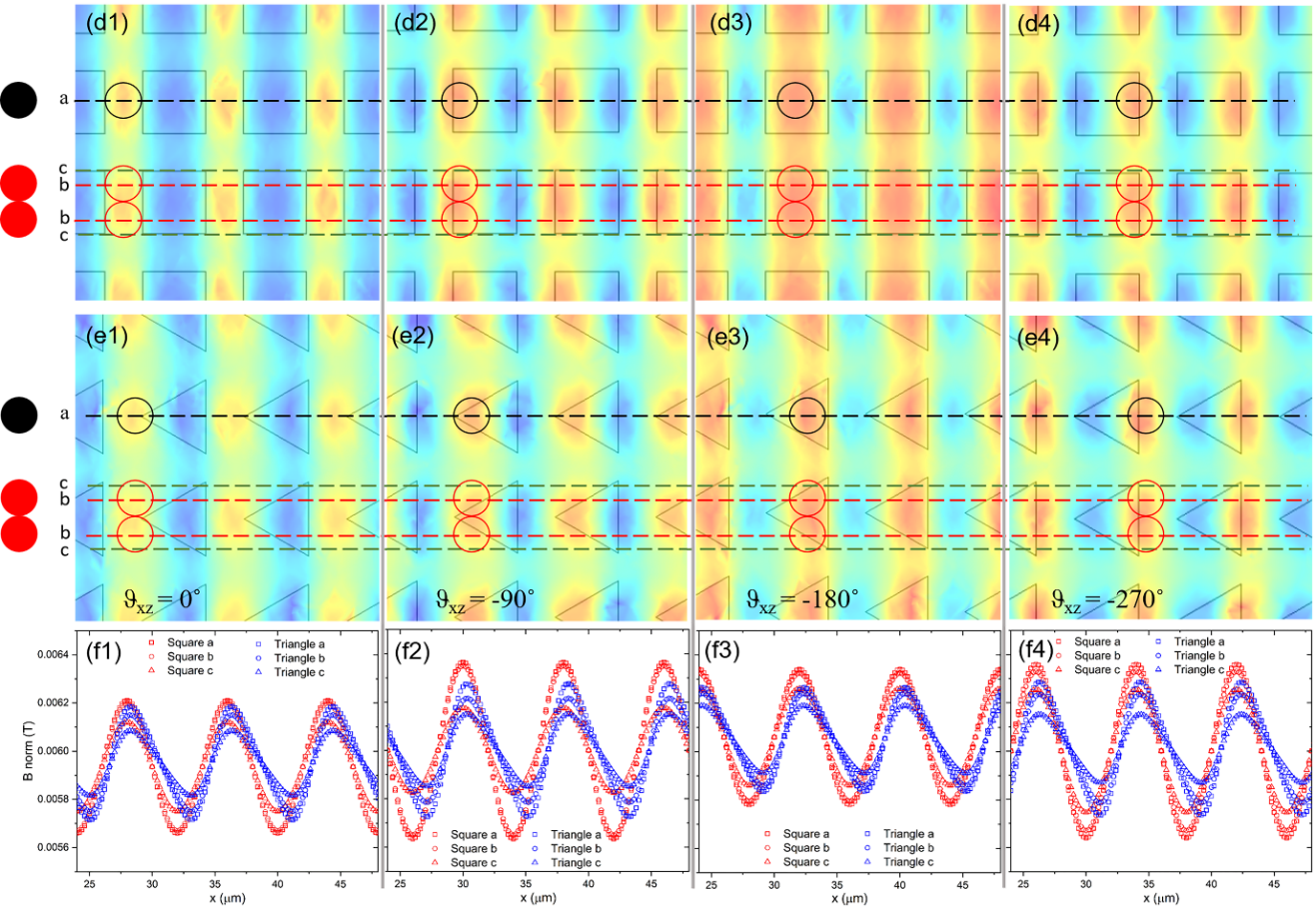


Figure S2 presents the FEM results for the magnetic flux density across a row of micromagnets in which the single SPM particle and dimer have been explicitly included. The FEM results have been presented for two orientations of the external magnetic field *θ_xz_* at -90^o^ and -270^o^. The results demonstrate the large and symmetric shape of the magnetic flux density on S for both single beads and dimer, as shown in Figure S2(a) and (b), respectively. The asymmetric shape of T shifted the beads’ position at *θ_xz_* = -90^o^, i.e., towards the center of the T micromagnet, as shown in Figure S2(c). At *θ_xz=_* -270^o^, the position of beads on T was almost aligned with that for S, due to similar magnitude of magnetic flux density (norm) generated by similar the dimensions of micromagnets in these positions. Figure S2(e) presents the magnetic flux density intensity in the y-direction across the SPM beads at an x-position that results in the perfect overlap curves at *θ_xz =_* -90^o^ and -270^o^. The magnitude of magnetic flux density on beads is slightly smaller for T, due to relative dimensions of the micromagnets. Particularly, the magnitude was lowest when the beads were positioned on the apexes of T at *θ_xz_* = -90^o^. This asymmetric feature of T appears to cause the relative larger differences in *ω_c_* between single beads and dimers.

**Figure S2. Analysis of the transport behavior of single beads and dimers on the square and triangular MMAs.** ) Results of FEM of the magnitude of magnetic flux density (norm) above (z=1.4 μm, i.e., in the center of 2.8 μm beads) NLM array surface at different *θ_xz_* for (a, b) square and (c, d) triangular shaped micromagnets. The magnitude of magnetic flux density along the vertical dash lines above the array are shown in figure (e). The dash lines are in the center of beads in y direction at z=1.4 μm.


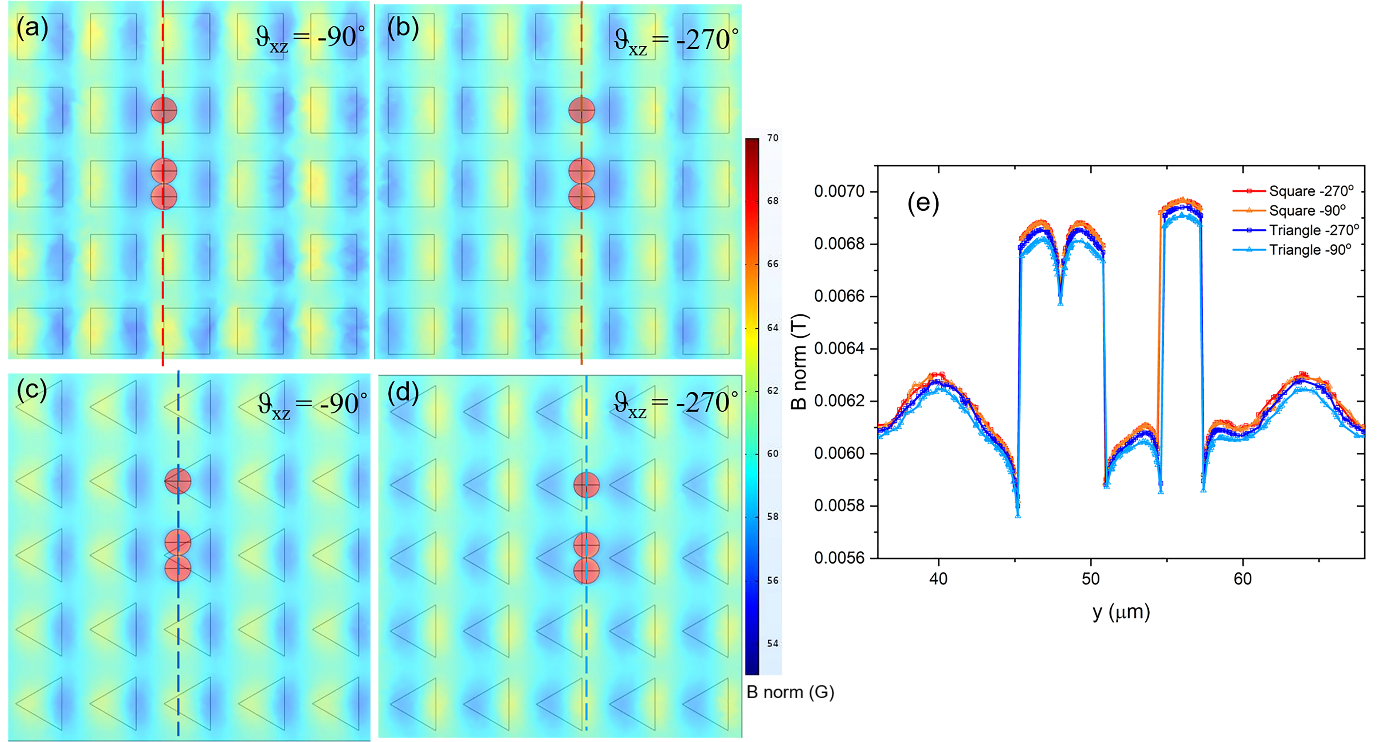

Supplement: Supplementary file 1 — Supplementary Information. [file 41598_2021_84395_MOESM1_ESM.docx]
